# Backpropagation Neural Network-Based Prediction Model of Marble Surface Quality Cut by Diamond Wire Saw

**DOI:** 10.3390/mi16090971

**Published:** 2025-08-23

**Authors:** Hui Dong, Fan Cui, Zhipu Huo, Yufei Gao

**Affiliations:** 1 Key Laboratory of High Efficiency and Clean Mechanical Manufacture of Ministry of Education, School of Mechanical Engineering, Shandong University, Jinan 250061, China; 2 School of Construction Machinery, Shandong Jiaotong University, Jinan 250357, China; 3 State Key Laboratory of Advanced Equipment and Technology for Metal Forming, Shandong University, Jinan 250061, China; 4 Shandong Key Laboratory of High Performance Tools and System, Shandong University, Jinan 250061, China

**Keywords:** marble, diamond wire sawing, surface quality, BP neural network, improved whale optimization algorithm

## Abstract

Marble is widely used in the field of construction and home decoration because of its high strength, high hardness and good wear resistance. Diamond wire sawing has been applied in marble cutting in industry due to its features such as low material loss, high cutting accuracy and low noise. The sawing surface quality directly affects the subsequent processing efficiency and economic benefit of marble products. The surface quality is affected by multiple parameters such as process parameters and workpiece sizes, making it difficult to accurately predict through traditional empirical equations or linear models. To improve prediction accuracy, this paper proposes a prediction model based on backpropagation (BP) neural network. Firstly, through the experiments of sawing marbles with the diamond wire saw, the datasets of surface roughness and waviness under different process parameters were obtained. Secondly, a BP neural network model was established, and the mixed-strategy-improved whale optimization algorithm (IWOA) was used to optimize the initial weight and threshold of the network, and established the IWOA-BP neural network model. Finally, the performance of the model was verified by comparison with the traditional models. The results showed that the IWOA-BP neural network model demonstrated the optimal prediction performance in both the surface roughness *Ra* and waviness *Wa*. The minimum predicted values of the root mean square error (RMSE), mean absolute error (MAE), and mean absolute percentage error (MAPE) were 0.0342%, 0.0284% and 1.5614%, respectively, which proved that the model had higher prediction accuracy. This study provides experimental basis and technical support for the prediction of the surface quality of marble material cut by diamond wire saw.

## 1. Introduction

As an important natural decorative material, marble is widely used in the field of architectural decoration, such as indoor and outdoor decoration, floor pavement, etc., because of its unique texture, rich colors, high compressive strength and good processability [1,2]. With the development of the high-end trend of marble decoration, the processing quality and cost are strictly controlled, which poses severe challenges to the processing technology [3].

The production process of marble slabs covers many procedures such as mining, cutting, grinding and polishing. The cutting process is particularly critical, because it directly determines the feasibility and cost of subsequent processing [4]. Traditional marble cutting methods such as circular sawing and frame sawing have many problems such as low processing accuracy, environmental pollution, high energy consumption and serious material waste [5,6,7,8]. Diamond wire sawing technology is gradually replacing the traditional processing methods and becoming an advanced method for cutting marbles due to its advantages such as low material loss, high processing accuracy, less dust and low energy consumption [9]. Diamond wire sawing uses wires with diamond abrasive grains consolidated on their surfaces as cutting tools. And the driving wheels drive the wire to make high-speed reciprocating or unidirectional movement, while the workpiece is fed toward the diamond saw wire at a certain speed through the feed system. Diamond-abrasive particles come into contact with the material and remove it through extrusion and scratching [10].

As a typical hard and brittle material, marble has high hardness, low plasticity and weak impact resistance, and it is prone to brittle failure. It often leads to poor surface quality during the cutting process. Therefore, in the research of cutting marble, relevant scholars still focus on the surface quality of marble slices. Yan et al. [11] conducted theoretical analysis and experimental research on different cutting forces and processing parameters during the milling of natural marble, established mathematical models and empirical equations, and analyzed the influences on material removal efficiency and surface quality. Yin et al. [12] conducted a white marble milling experiment using the Box–Behnken design method of the response surface method (RSM), established a multiple nonlinear regression model, and analyzed the influence of various processing parameters on the processing quality of white marble. Pham et al. [13] studied the influence of cutting parameters on the surface roughness of marble processed by diamond saw blade. They conducted a regression analysis using experiments and an analysis of variance. The results indicated that the cutting parameters had a significant impact on surface roughness. Dong et al. [14] studied the sawing performance and blade instability of diamond frame saw in granite processing through sawing experiments. The research found that long contact arcs and continuous long thin chips would lead to the instability of the saw blade, thereby reducing the slice flatness. Liu et al. [15] studied the influence of parameters such as wire speed on the surface quality of marble when a circular electroplated diamond wire saw was used to cut marble through orthogonal experiments, and optimized the process parameters. In summary, the surface quality of marble slices is influenced by various parameters such as process parameters and workpiece size parameters, which makes it difficult to accurately predict the surface quality through traditional empirical equations or linear models. In the actual production, it is necessary to adjust the process parameters through repeated trial cutting, which makes it hard to ensure the stability of processing quality [16,17]. Moreover, there are currently few studies on the surface quality prediction of marble cut by diamond wire saws. Therefore, establishing a model that can accurately predict the surface quality of marble cut by diamond wire saws is of great significance and practical application value for achieving the optimal configuration of processing parameters, improving production efficiency, reducing costs and ensuring product quality.

In recent years, a variety of innovative methods have been developed for predicting the surface quality of machining. For example, Eaysin et al. [18] applied models such as support vector regression (SVR) and gradient boosting regression tree (GBRT) to predict the removal rate and surface roughness of W70Cu30 material. Guo et al. [19] integrated a convolutional neural network (CNN) and multi-head self-attention (MHA) model and proposed a surface roughness prediction method based on a hybrid neural network. Huang et al. [20] developed a combined prediction system for two materials based on artificial neural networks (ANNs). Liu et al. [21] established a surface roughness prediction model with strong generalization ability by using a backpropagation (BP) neural network optimized by genetic algorithm (GA-BP). Research has found that many scholars have conducted extensive work in the field of surface prediction, and prediction methods have evolved from traditional empirical formulas and linear regression methods to data-driven nonlinear model methods.

In particular, a BP neural network has been widely used in the field of industrial prediction due to its powerful self-learning and adaptability [22,23]. A BP neural network induces implicit rules in data through self-training and adjustment, and can capture the complex correspondence between the input and output. However, the BP neural network also has some limitations, such as weak global search ability and being prone to getting stuck in local extremums, resulting in insufficient prediction accuracy [24,25]. To solve these problems, researchers proposed to use intelligent optimization algorithms to optimize BP neural networks to improve global optimization capabilities and prediction accuracy [26]. The whale optimization algorithm (WOA), as a novel swarm intelligence optimization method, simulates the hunting behavior of humpback whales and demonstrates strong search potential. It has been applied in multiple fields such as neural network training and engineering optimization [27,28]. However, when dealing with complex optimization problems, WOA has some limitations such as lagging convergence speed and insufficient local extremum escape capability. In recent years, researchers have significantly improved the performance of WOA by improving the spiral update position model, introducing elite reverse learning strategies, chaotic mapping and other methods [29,30,31]. For example, An et al. [32] introduced particle swarm optimization (PSO) to optimize a BP neural network and predict the cutting quality of silicon wafers. The results showed that the possibility of getting stuck in local minimum was significantly reduced. Wu et al. [33] proposed an improved whale optimization algorithm (IMWOA), which significantly enhanced the convergence accuracy and speed of the algorithm by optimizing the spiral update position model and combining multiple strategies, as well as its stability and ability to solve ultra-large-scale optimization problems. The improved whale optimization algorithm (TIWOA) proposed by Huang [34] significantly improved the global search ability and convergence speed of the algorithm by optimizing initialization strategy and introducing mutation operator and other improvement measures. Ji et al. [35] proposed a prediction model based on the improved whale optimization algorithm (CIWOA) combined with a BP neural network, which significantly enhanced the prediction accuracy and stability. These studies show that the improved whale optimization algorithm can more effectively optimize the parameters of the BP network and enhance its prediction performance.

In this paper, *Ra* and *Wa* were taken as the surface quality parameters of the slices. Through single-factor experiments, orthogonal experiments and response surface experiments of cutting marble with diamond wire saw, surface quality datasets under different process parameters were obtained. The whale optimization algorithm (IWOA) improved by the hybrid strategy was adopted to optimize the BP neural network model, and the improved IWOA-BP neural network model was established. The performance of the model was verified by comparing it with the traditional models. The research results provide more reliable theoretical support and technical reference for the intelligent optimization of the cutting process parameters of diamond wire saw, and offer important practical guidance and reference value for actual processing.

## 2. Materials and Methods

### 2.1. Experimental Materials

**Figure 1:**
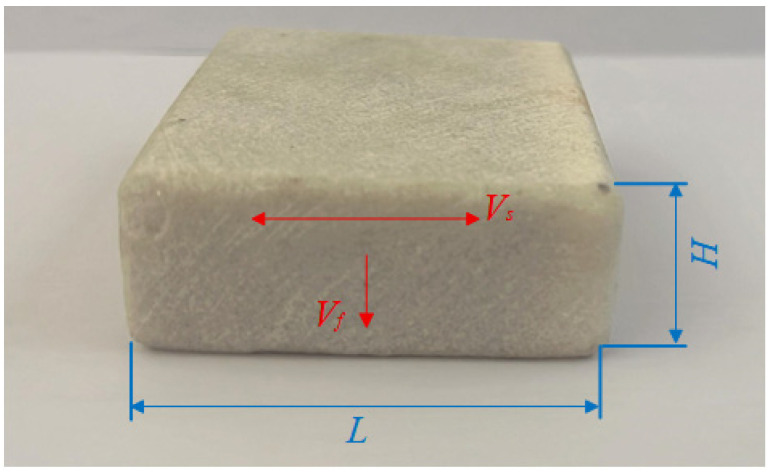
Experimental marble sample.

The experiments took marble as the research object. Its main chemical component is calcium magnesium carbonate (CaMg(CO_3_)_2_), and the mineral composition is mainly dolomite, with a small amount of mica and trace amounts of quartz and argillaceous impurities, as shown in Figure 1.

**Figure 2:**
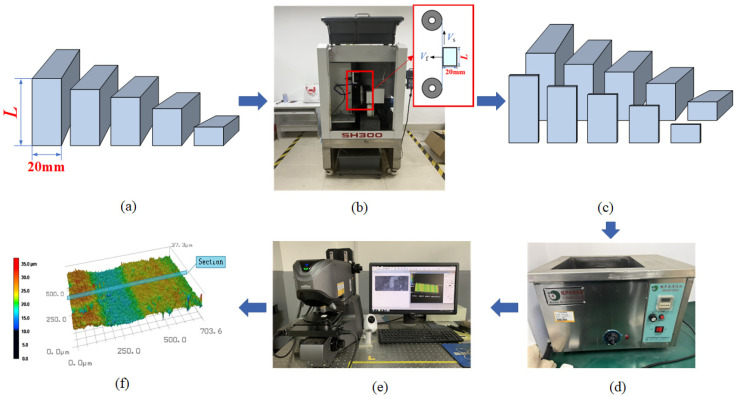
Experimental process diagram. (**a**) Marble workpieces for experiment; (**b**) wire sawing; (**c**) marble slices; (**d**) ultrasonic cleaning; (**e**) observation by laser confocal microscopy; (**f**) data processing.

**Figure 3:**
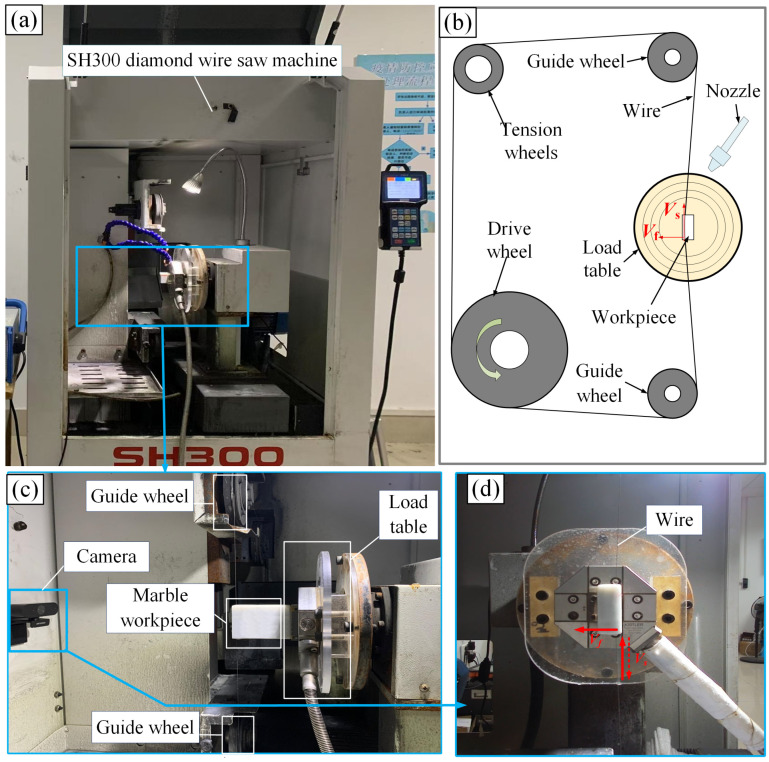
Experimental setup diagram. (**a**) Wire sawing equipment diagram; (**b**) wire sawing principle diagram; (**c**) main view of processing; (**d**) left view of processing.

The experimental process is shown in Figure 2. Firstly, the diamond wire sawing machine (SH300, Guangzhou Shenghai Electronic Technology Co., Ltd., Guangzhou, China) was used to cut the marble workpiece, and the experimental parameters are shown in Table 1. After ultrasonic cleaning, the surface morphology of the slices was observed using the VK-X200K laser confocal microscope (Keyence Corporation, Osaka, Japan), and quality indicators such as *Ra* and *Wa* were obtained. After the completion of data collection, combined with the sawing process parameters and the collected surface quality data, the neural network prediction model was established by using data analysis and modeling methods to achieve the accurate prediction of marble cutting surface quality. The cutting principle of the experiment is shown in Figure 3b. The workpiece was fixed on the loading platform. After the machine started, the wire saw moved back and forth at the wire speed *V_s_*. at the same time, the loading platform drove the workpiece to move at a constant feed speed *V_f_* to complete the sawing. The installation of the marble sample in the sawing experiment is shown in Figure 3c,d.

### 2.2. Experimental Methods

To establish an effective prediction model, this study collected data by designing sawing experiments. The experimental design included single factor experiments, orthogonal experiments and response surface experiments. The experiment results together formed the dataset for model training and testing. Previous research indicated that the feed speed *V_f_* and wire speed *V_s_* of diamond wire saw had a significant impact on the surface *Ra* of the sawn surface [36,37,38,39]. In addition, the variation in the length *L* of the sawing would affect the cooling, lubrication and chips removal during the sawing, and influence the quality of the sawed surface [40,41]. Therefore, in this paper, the feed speed *V_f_*, wire speed *V_s_*, and sawing length *L* were selected as three experimental factors for experimental design. After the slicing was completed, each group of slices was cleaned and observed. Five positions were randomly selected in the central area of each slice to measure the *Ra* and *Wa*, and the average value was taken as the characterization index.

The single factor experiments can reflect the influence of a single sawing factor on the surface quality of marble. Each experimental parameter factor selected for the experiment was set at five levels. A total of 13 parameter combinations were selected after removing duplicates, as shown in numbers 1 to 13 in Table 2.

The orthogonal experiment also set five levels for each factor, forming a set of three-factor and five-level orthogonal experiments. After removing the duplicates, a total of 24 parameter combinations were selected, as shown in numbers 14 to 37 in Table 2.

The response surface experiments were designed based on the Box-Behnken design (BBD) response surface experiment, with each factor set at low, medium, and high levels respectively. This level setting not only comprehensively covered the influence range of each factor on *Ra* and *Wa*, but also fully reflected the interaction among the factors. Based on the above level settings and the characteristics of the BBD design, 10 parameter combinations were selected. Each combination contained the interaction of three levels, as shown in numbers 38 to 47 in Table 2. Table 2 summarizes all the parameter combinations of the sawing experiments; the repetitive parameter combinations in the three experimental designs were removed.

### 2.3. Evaluation Method of Slices Surface Quality

**Figure 4:**
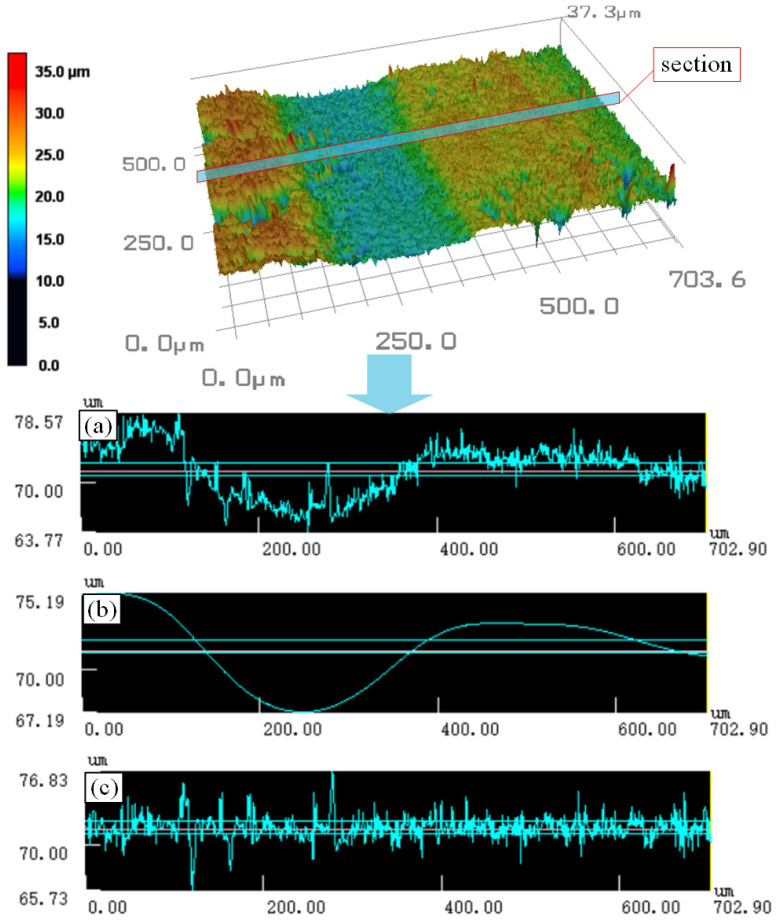
Three-dimensional topography of the slice surface and the data measurement diagram. (**a**) Cross-sectional contour curve; (**b**) waviness curve; (**c**) roughness curve.

As shown in Figure 4, after obtaining the three-dimensional surface topography images of the slices using the laser confocal microscope, detailed measurements and analyses were conducted on the *Ra* and *Wa*. Figure 4a–c, respectively, presents the cross-sectional contour curve, waviness curve, and roughness curve. The calculation equation of the waviness profile arithmetic mean deviation is as follows [41]:

(1) $$Wa=\frac{1}{l_{w}}\int_{0}^{l_{w}}\left|Z(x)\right|dx$$

where *l_w_* is the sampling length of waviness, and *Z*(*x*) is the distance between the point on the waviness contour and the centerline of waviness.

### 2.4. Experimental Results

The experimental data were sorted out, as shown in Table 3. This dataset covered all the sawing parameter combinations designed in this study and their corresponding *Ra* and *Wa* values, which were used for the training and prediction of neural networks.

## 3. Surface Quality Prediction Model of Diamond Wire Sawing Marble Based on the IWOA-BP Neural Network

### 3.1. The Structure and Parameter Selection of a BP Neural Network

**Figure 5:**
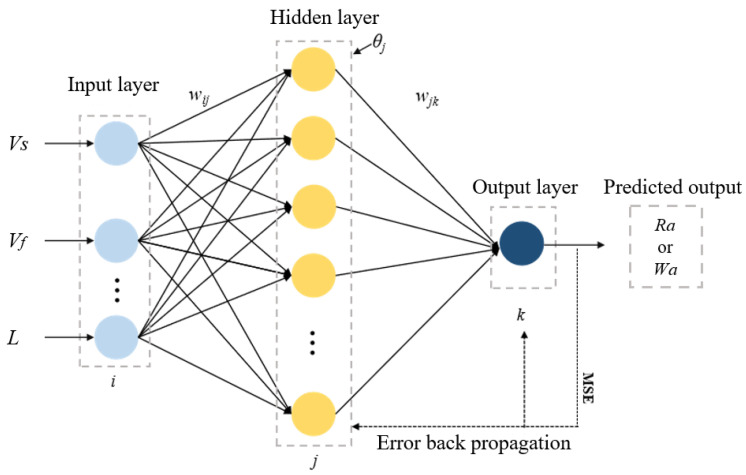
Structure diagram of the three-layer BP neural network.

A BP neural network is a multi-layer feedforward neural network based on the error backpropagation algorithm, consisting of an input layer, a hidden layer and an output layer. Information is passed layer by layer from the input layer to the output layer, and the prediction results are generated through weight connection and activation function processing. The network optimizes performance by calculating the output error and backpropagating the weights and thresholds [42]. Hornik et al. [43] discovered that by choosing the appropriate activation function and the number of hidden layer nodes, a three-layer neural network can approximate any function. Therefore, this paper adopted a three-layer neural network structure to establish a prediction model, and the network structure is shown in Figure 5.

After determining the structure of the BP neural network, the parameters of the neural network were set. Through the previous analysis, the influencing factors of the surface quality of marble slices cut by diamond wire saw included the feed speed, wire speed and sawing length. Therefore, the number of input layer nodes was set to 3. The number of output layer nodes was set to 1, and the *Ra* and *Wa* were separately trained and predicted. According to the empirical Equation (2), it could be determined that the selection range of the number of nodes was [3,12]. This paper determined and selected the best number of hidden layer nodes by calculating RMSE value of the model corresponding to different hidden layer nodes, and applied it to the subsequent training and prediction.

(2) $$h=\sqrt{m+n}+\xi$$

where *m*, *h* and *n* are the number of nodes in the input layer, hidden layer and output layer, respectively. It is a regulating constant and the value range is between 1 and 10.

Activation function is the output function of neurons, which enables neural networks to learn and fit complex function relationships. Sigmoid function has smooth gradient and good derivative properties, so it was used as the activation function of the hidden layer. In this paper, the learning rate was selected as 0.01, the error objective function of the model was the mean square error (MSE), the training target accuracy was set as 0.00001, and the maximum training times was set as 1000 times.

### 3.2. Whale Optimization Algorithm Improved by Hybrid Strategy

Although WOA has a strong optimization ability, it constantly changes the position of the population leader in the search space, which can easily lead to premature convergence of the algorithm when it gets stuck in the local optimal solution, thereby affecting the global optimization performance [44]. To enhance the optimization capability of WOA, the following improvements were made to WOA in this section:

(1). Population initialization based on sine chaotic mapping and quasi-reverse learning strategy:

WOA initializes the population individuals using a random method, which may lead to an uneven initial distribution of the population and thereby affect the optimization accuracy. To solve this problem, this paper introduced sine chaotic initialization and quasi-inverse learning initialization. These two methods could generate initial feasible solutions with good diversity, and their equation is as follows:

(3) $$x_{n+1}=\frac{\beta }{4}\sin(\pi x_{n})$$

where *x_n_* is the *n*-th chaotic number and *n* is the number of iterations in sine chaotic map. *β* is a randomly selected constant. In this paper, *β* is selected as 4.

To further improve the quality of the initial population, a quasi-reverse learning strategy was added on the basis of the sine chaotic mapping to enhance the distribution uniformity and exploration ability of the population. Suppose the size of the whale population is N and the search space is q-dimensional, then the position of the *i*-th whale could be expressed as $X_{i}=$ ($X_{i}^{1}$, $X_{i}^{2}$, $X_{i}^{3}$, ⋯, $X_{i}^{q}$) (*i* = 1, 2, ⋯, *N*), where the value of each position coordinate $X_{i}^{j}$ (*j* = 1, 2, ⋯, q) is between [$c_{i}^{j}$, $d_{i}^{j}$], $c_{i}^{j}$ and $d_{i}^{j}$ represent the lower and upper bounds of $X_{i}^{j}$ respectively. The mathematical equations of quasi-inverse solution are as follows:

(4) $${\hat{X}}_{i}^{j}=\left\{\begin{array}{ll} rand(avg_{i}^{j},{\hat{X}}_{i}^{j}) & X_{i}^{j}\leq avg_{i}^{j}\\ rand({\hat{X}}_{i}^{j},avg_{i}^{j}) & X_{i}^{j}>avg_{i}^{j} \end{array}\right.$$

(5) $$avg_{i}^{j}=\frac{d_{i}^{j}-c_{i}^{j}}{2}$$

(2). Nonlinear convergence factor improvement strategy:

In original WOA, it is assumed that the optimal individual in the current population represents the prey, and other whale individuals in the population then surround the position of the optimal individual to update their own positions. This position update process is achieved through Equations (6) and (7):

(6) $$D=|C\cdot X^{*}(t)-X(t)|$$

(7) $$X(t+1)=X^{*}(t)-A\cdot D$$

where *D* represents the distance between the current individual and the optimal solution. *t* represents the number of iterations. *A* and *C* are coefficient vectors. X*(*t*) is the optimal position vector of the population when iterating to the *t*-th generation. *X*(*t*) is the position vector of the current population.

In the process of optimization, WOA will face the problem of imbalance between global search ability and local development ability [28]. In WOA, the parameter |*A*| determines the switch between global search and local development. When |*A*| ≥ 1, the algorithm will perform random global search; when |*A*| < 1, the algorithm will develop locally. The trend of the system variable *A* is affected by the linear convergence factor *a*. In this paper, an improved nonlinear convergence factor was proposed, and its calculation equation is as follows:

(8) $$a=2e^{-\frac{t}{tau}}$$

(9) $$tau=\frac{Max\_iter}{2}$$

where *Max_iter* is the maximum number of iterations. Based on the improved nonlinear convergence factor, the update equation of the system variable *A* becomes

(10) $$A=(4r_{1}-2)e^{-\frac{t}{tau}}$$

The equation for the coefficient vector *C* is as follows:

(11) $$C=2r_{2}$$

where *r*_1_ and *r*_2_ are random numbers within the range of [0, 1].

**Figure 6:**
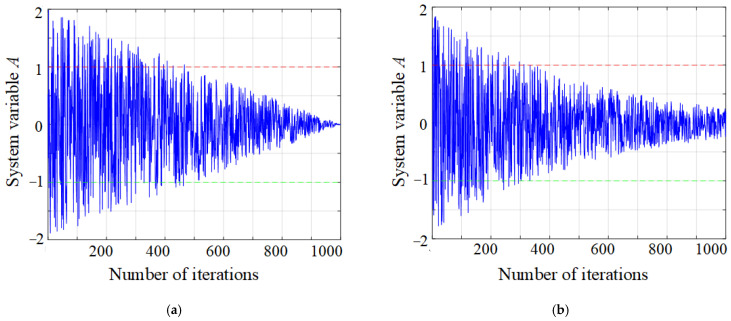
Comparison of system variable *A* before and after improvement. (**a**) Before improvement; (**b**) after improvement.

The changing trend of system variable *A* with the number of iterations before and after improvement is shown in Figure 6. The time when the improved system variable *A* enters the interval [−1, 1] is earlier than that of the unimproved *A*, and the rate of decline in the later stage is slower. This indicates that the improved *A* has a strong global search ability in the early stage, more stable local development in the later stage, it can better balance the global and local aspects.

(3). Adaptive weighting strategy:

WOA did not have the function of adjusting weights through updating equations during later partial development. With the deepening of the search, individual whales may wander around the theoretical position, or even fall into local extremum. Therefore, this paper used the concept of inertia weight in particle swarm optimization algorithm for reference, and applies the adaptive weight *w* to WOA to optimize the search process. The calculation equation is as follows:

(12) $$w=w_{\max}+(w_{\max}-w_{\min})\cdot \sin(\pi +\frac{\pi t}{2Max\_iter})$$

where *w*_min_ = 0.4 and *w*_max_ = 0.9, and the values refer to the common parameter settings in particle swarm optimization algorithm.

**Figure 7:**
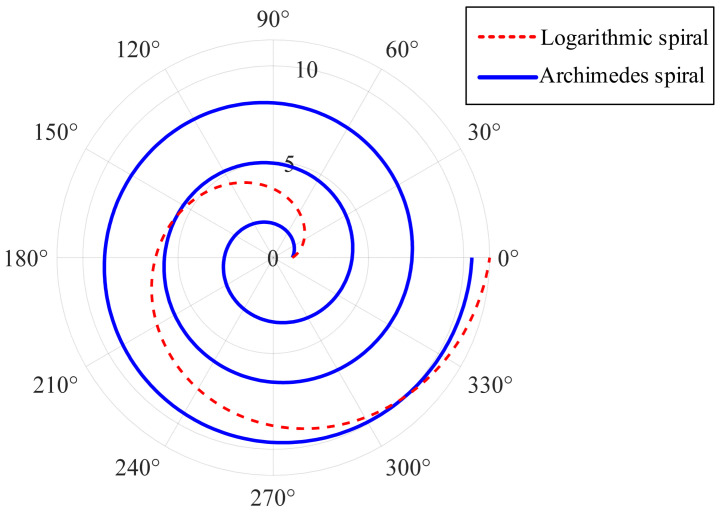
Comparison of the polar coordinate diagrams of the logarithmic spiral and the archimedian spiral.

Therefore, the position update strategy of the algorithm is adjusted as follows:

(13) $$X(t+1)=\left\{\begin{array}{ll} w\cdot X^{*}(t)-A\cdot D & p<0.5\\ D\cdot e^{bl}\cdot \cos(2\pi l)+w\cdot X^{*}(t) & p\geq 0.5 \end{array}\right.$$

where *b* represents a constant in the spiral motion equation of the whale herd. It is a constant and is usually set to 1. *l* is a random number with the range [−1, 1]. *p* is a random number within the range [0, 1]. In this paper, *p* was selected as 0.5, which can not only maintain the global search ability but also avoid falling into local optimum too early [29].

(4). Improved spiral position update strategy:

The original WOA algorithm uses the logarithmic spiral to update the position model in the hunting stage, which is easy to make the population gather. Therefore, this paper replaced the logarithmic spiral in the original equation with the archimedian spiral, and the polar image comparison is shown in Figure 7. It can be seen that the archimedian spiral can effectively reduce the step interval of individual whales, expand the search range, and avoid algorithm premature convergence. Combined with the adaptive weight strategy, the new spiral position update model is shown in Equation (14).

(14) $$X(t+1)=D\cdot (bl)\cdot \cos(2\pi l)+w\cdot X^{*}(t)$$

(5). Random differential mutation strategy:

Using the idea of differential evolution algorithm for reference, the current whale individual, the optimal individual and other randomly selected individuals are used for differential calculation. After each iteration, the population is disturbed to generate new individuals, so as to enhance the diversity of the population and avoid search stagnation. The mathematical equation is as follows:

(15) $$X(t+1)=\rho_{1}\cdot (X^{*}(t)-X(t))+\rho_{2}\cdot (X^{\prime }(t)-X(t))$$

where *ρ*_1_ and *ρ*_2_ are random numbers within the range [0,1], and *X*’(*t*) is the individual whale randomly selected from the population.

**Figure 8:**
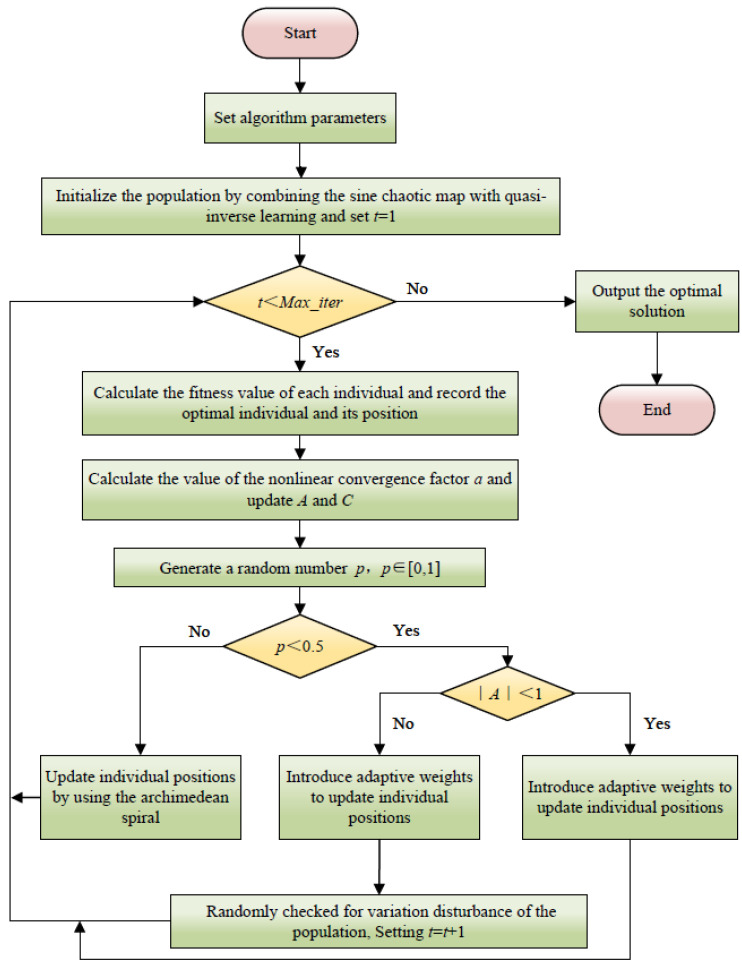
Flowchart of the IWOA algorithm.

Combining the above five hybrid strategies to improve WOA, the improved algorithm is called IWOA (improved whale optimization algorithm), and its flowchart is shown in Figure 8.

### 3.3. Algorithm Performance Testing and Result Analysis

**Figure 9:**
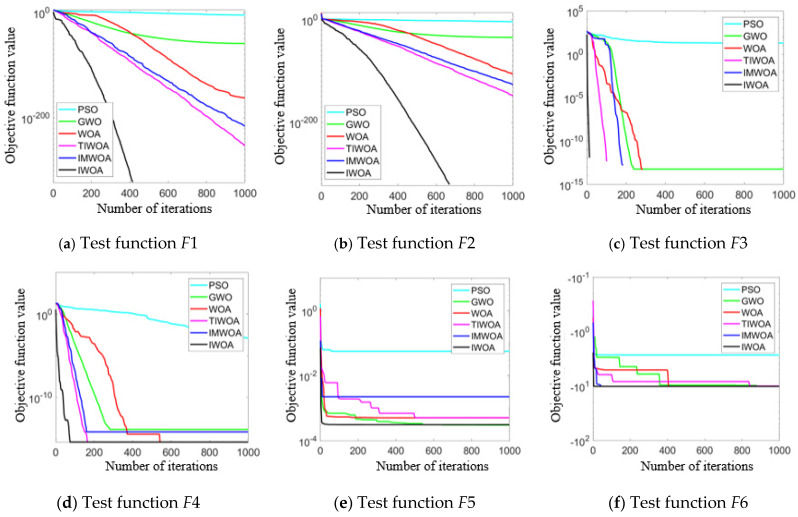
Algorithm convergence curve.

(1). Parameter settings and test function sets:

To verify the superiority of IWOA performance, this paper conducted performance comparison tests with algorithms include PSO, GWO (Grey Wolf Optimizer), WOA, TIWOA and IMWOA. In the experiment, all algorithms adopted the same general condition settings, with a population size of 30 and a maximum number of iterations of 1000. This paper selected six commonly used test functions [29] for testing. The specific functions are shown in Table 4.

(2). Comparative analysis of algorithm convergence curves:

The algorithm convergence curves of the six test functions are shown in Figure 9, where the horizontal axis represents the number of iterations and the vertical axis represents the fitness objective function value. The smaller the value, the better the optimization performance of the algorithm. It can be seen from the results that IWOA demonstrated significant advantages on almost all test functions and was clearly superior to other algorithms. IWOA could quickly obtain a relatively low fitness value in the early stage of iteration and continuously optimize it in subsequent iterations, eventually convergent to a better objective function value. It indicates that IWOA has a superior convergence speed and global search ability compared to other algorithms.

(3). Comparison of test results of each algorithm:

To avoid deviations in a single result, 30 independent simulation tests were conducted for each test function to obtain the best value (Best), standard value (Std), and average value (Avg) of the objective function. The Best, Std and Avg, respectively, reflect the best performance, stability and average performance of the algorithm in multiple runs. In this section, all the test functions are minimization problems. Therefore, the smaller the values of these indicators, the better the performance of the algorithm. The optimization results of the six different algorithms are shown in Table 5. The numbers marked in black bold in the table are the best results. The analysis of the data show that among the six groups of test functions, all the indicators calculated by IWOA are superior to those of the other algorithms. Especially in the *F*1 and *F*3 test functions, the IWOA have reached the theoretical optimal solutions of the test functions, which fully demonstrates that IWOA has higher convergence accuracy and stronger robustness.

### 3.4. The IWOA Algorithm Optimizes BP Neural Networks

**Figure 10:**
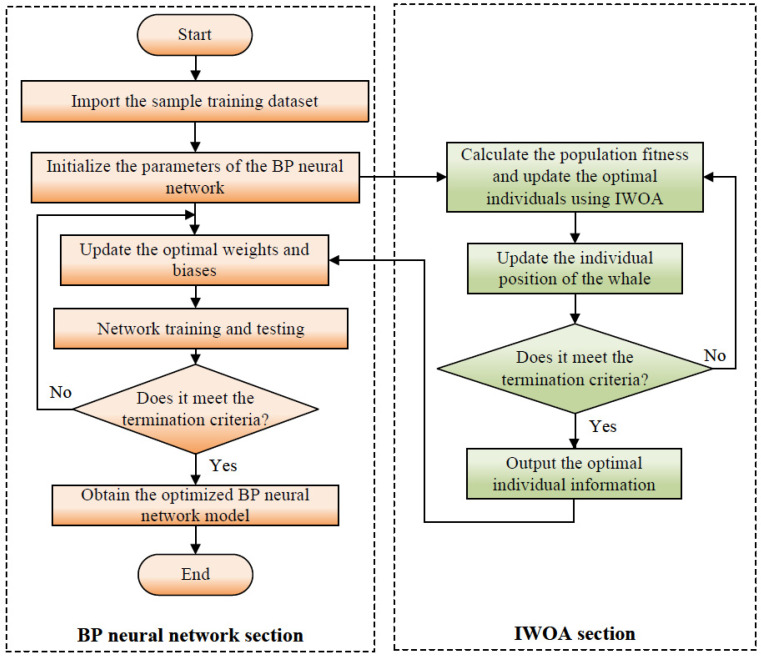
Flowchart of the IWOA-BP algorithm.

Although a BP neural network has strong fitting ability, it is easy to getting stuck in local extremum and has weak global search ability [44]. The improved whale optimization algorithm (IWOA) has a powerful global search capability, which can effectively avoid local optima and quickly converge to the global optimal solution. Therefore, combining IWOA with a BP neural network and using IWOA to optimize the initial weights and thresholds of the BP neural network can enhance the prediction accuracy and generalization ability of the model. This combined model is called the IWOA-BP neural network model, and its flowchart is shown in Figure 10.

### 3.5. Comparison of Neural Network Prediction Effects

To verify the prediction accuracy of the IWOA-BP neural network model, this paper conducted a comparative analysis with the traditional BP neural network, included the WOA-BP, TIWOA, TIWOA-BP and IMWOA-BP neural networks. The fitness function of neural networks is the root mean square error. Based on the dataset organized in Table 3, 10 groups were randomly selected as the test set, and the remaining 37 groups of data are used as the training set. The numbers marked with “a” in Table 3 represent the test set data of the model. The training and testing of these five neural network models were completed. The gap between the predicted values and the true values on the test set was compared, and the prediction errors were calculated to evaluate the prediction accuracy of each model.

**Figure 11:**
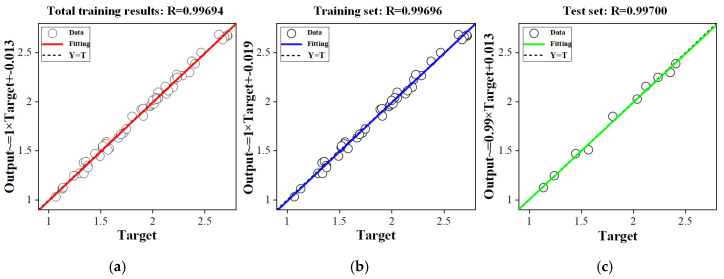
Training results of neural network model. (**a**) Total training results; (**b**) training set results; (**c**) test set results.

Taking the *Wa* as an example, the neural network model had three nodes in the input layer and one node in the output layer. In the training process, RMSE corresponding to the number of nodes in different hidden layers was compared after multiple training, and the optimal number of nodes in hidden layers was selected. As shown in Table 6, when the number of hidden layer nodes is 6, RMSE is the smallest (0.0342), so 6 was selected as the optimal number of hidden layer nodes. In addition, the effect of the model was evaluated by correlation analysis. As shown in Figure 11, the correlation coefficient *R* of the training set, test set and total set were all greater than 0.99, indicating that the model had a high fitting degree and ideal training effect. The neural network model was saved for subsequent prediction. The establishment process of the neural network prediction model of *Wa* was the same as that of *Ra*.

**Figure 12:**
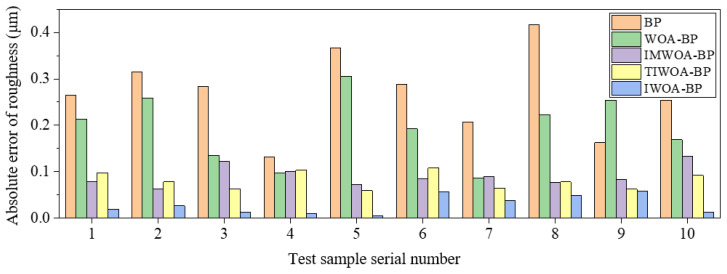
Comparison of absolute errors in *Ra* prediction by different neural network models.

**Figure 13:**
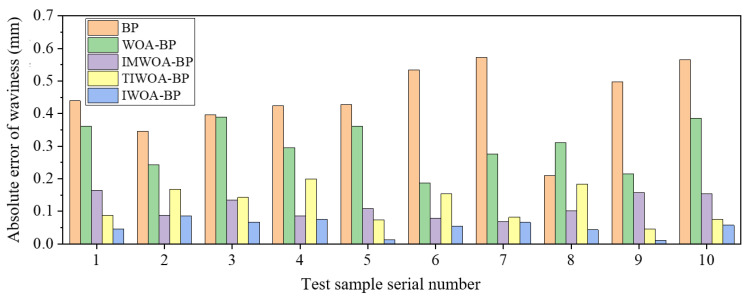
Comparison of absolute errors in the prediction of *Wa* by different neural network models.

BP, WOA-BP, IMWOA-BP, TIWOA-BP and IWOA-BP neural networks were used to train and predict the experimental data of *Ra* and *Wa*, and the prediction results were compared and analyzed. Table 7 and Table 8 show the prediction results of these five models. To present the differences among various models more intuitively, the columnar comparison diagrams of the prediction absolute errors of *Ra* and *Wa* by different neural network models are drawn, as shown in Figure 12 and Figure 13. It can be seen that the predicted values of the IWOA-BP model were the closest to the true values, and absolute prediction errors were significantly lower than that of other models. This indicates that the IWOA-BP model performs well in the prediction of surface *Ra* and *Wa* of marble cut by diamond wire saw in this paper, and has higher accuracy.

To comprehensively evaluate the regression prediction performance of the optimized neural network model, this paper adopted three commonly used prediction performance evaluation indicators: RMSE, MAE and MAPE. These three indicators could reflect the accuracy of the model’s prediction results from different perspectives, avoiding the limitations brought by a single indicator.

The test results are shown in Table 9. The *Ra* prediction results showed that the RMSE, MAE and MAPE values of the IWOA-BP neural network model were 0.0342, 0.0284 and 1.5614%, respectively, all of which were lower than the corresponding error values of the other four models. The *Wa* prediction results showed that the RMSE, MAE and MAPE of the IWOA-BP neural network model were 0.0570, 0.0520 and 1.7028%, respectively, which were also lower than the corresponding error values of the other four models. This indicated that the IWOA-BP neural network model demonstrated the optimal prediction performance in both *Ra* and *Wa* predictions.

## 4. Conclusions

This paper established an IWOA-BP neural network model for predicting the surface quality of marble cut by diamond wire saw based on the BP neural network model optimized by the IWOA. Through single factor experiments, orthogonal experiments and response surface experiments, the datasets of *Ra* and *Wa* under different sawing parameters were collected systematically. The results were used for model training and testing. Finally, the performance of various models was compared. The research reached the following conclusions:

(1). Through multiple trainings on *Ra*, the RMSE corresponding to different numbers of hidden layer nodes was compared. When the number of hidden layer nodes is 6, the RMSE is the smallest (0.0342). The correlation coefficients of the training set, test set, and total set were all greater than 0.99, indicating that the model had a high degree of fitting and an ideal training result.
(2). The comparison of the prediction results showed that the *Ra* prediction error (RMSE = 0.0342, MAE = 0.0284, MAPE = 1.5614%) and *Wa* prediction error (RMSE = 0.0570, MAE = 0.0520, MAPE = 1.7028%) of the IWOA-BP neural network were all lower than those of the traditional BP, WOA-BP and IMWOA-BP and IMWOA-BP neural network models.

The results show that the IWOA-BP neural network model has certain advantages in predicting the surface quality of marble cut by diamond wire saw. The research results not only enrich the prediction method of cutting surface quality of hard and brittle materials, but also provide a theoretical basis and practical reference for the surface quality control of diamond wire sawing.

## Figures and Tables

**Table 1:** Parameters of the wire sawing and workpiece.

Parameter	Value
Saw wire type	Nickel-plated diamond wire
The diameter of the saw wire core (μm)	220
Abrasive particle size (μm)	25–35
Particle distribution density (grits/mm^2^)	70–85
Slice thickness (mm)	2
Sawing length *L* (mm)	10, 20, 30, 40, 50
Workpiece thickness *H* (mm)	20

**Table 2:** Parameter combinations.

No.	Feed Speed, *V_f_* (mm/min)	Wire Speed, *V_s_* (m/min)	Sawing Length, *L* (mm)
1	1.8	1000	10
2	1.8	1000	20
3	1.8	1000	30
4	1.8	1000	40
5	1.8	1000	50
6	1.8	600	30
7	1.8	800	30
8	1.8	1200	30
9	1.8	1400	30
10	0.6	1000	30
11	1.2	1000	30
12	2.4	1000	30
13	3	1000	30
14	0.6	600	10
15	0.6	800	30
16	0.6	1000	50
17	0.6	1200	20
18	0.6	1400	40
19	1.2	600	50
20	1.2	800	20
21	1.2	1000	40
22	1.2	1200	10
23	1.2	1400	30
24	1.8	600	40
25	1.8	800	10
26	1.8	1200	50
27	1.8	1400	20
28	2.4	600	30
29	2.4	800	50
30	2.4	1000	20
31	2.4	1200	40
32	2.4	1400	10
33	3	600	20
34	3	800	40
35	3	1000	10
36	3	1200	30
37	3	1400	50
38	0.6	600	30
39	3	600	30
40	0.6	1400	30
41	3	1400	30
42	0.6	1000	10
43	3	1000	50
44	1.8	600	10
45	1.8	1400	10
46	1.8	600	50
47	1.8	1400	50

**Table 3:** Experimental data.

No.	Feed Speed, *V_f_* (mm/min)	Wire Speed, *V_s_* (m/min)	Sawing Length, *L* (mm)	Roughness, *Ra* (μm)	Waviness, *Wa* (μm)
1 ^a^	1.8	1000	10	1.445	2.527
2	1.8	1000	20	1.683	2.921
3	1.8	1000	30	1.952	3.278
4	1.8	1000	40	2.081	3.516
5 ^a^	1.8	1000	50	2.236	3.783
6	1.8	600	30	2.226	3.684
7	1.8	800	30	2.035	3.436
8 ^a^	1.8	1200	30	1.802	3.115
9	1.8	1400	30	1.723	3.001
10	0.6	1000	30	1.35	2.054
11	1.2	1000	30	1.667	2.746
12	2.4	1000	30	2.097	3.674
13	3	1000	30	2.267	4.037
14	0.6	600	10	1.271	1.735
15	0.6	800	30	1.39	2.167
16 ^a^	0.6	1000	50	1.568	2.45
17	0.6	1200	20	1.113	1.71
18	0.6	1400	40	1.329	2.246
19	1.2	600	50	2.278	3.561
20	1.2	800	20	1.571	2.207
21	1.2	1000	40	1.854	2.988
22 ^a^	1.2	1200	10	1.136	1.961
23	1.2	1400	30	1.446	2.532
24	1.8	600	40	2.414	3.979
25	1.8	800	10	1.535	2.721
26	1.8	1200	50	2.112	3.572
27	1.8	1400	20	1.523	2.583
28 ^a^	2.4	600	30	2.354	4.158
29	2.4	800	50	2.663	4.618
30 ^a^	2.4	1000	20	2.036	3.66
31	2.4	1200	40	2.148	3.798
32	2.4	1400	10	1.378	2.525
33	3	600	20	2.503	4.097
34	3	800	40	2.68	4.603
35	3	1000	10	1.632	3.108
36 ^a^	3	1200	30	2.12	3.862
37 ^a^	3	1400	50	2.407	4.267
38	0.6	600	30	1.548	2.323
39	3	600	30	2.672	4.569
40 ^a^	0.6	1400	30	1.24	1.895
41	3	1400	30	2.041	3.701
42	0.6	1000	10	1.032	1.536
43	3	1000	50	2.685	4.63
44	1.8	600	10	1.59	2.735
45	1.8	1400	10	1.269	2.239
46	1.8	600	50	2.634	4.305
47	1.8	1400	50	2.014	3.441

^a^ Test set data.

**Table 4:** Test functions.

Expression	Dimension	Initial Range	Theoretical Minimum Value
$F_{1}(x)=\sum_{i=1}^{n}x_{i}^{2}$	30	[−100, 100]	0
$F_{2}(x)=\sum_{i=1}^{n}|x_{i}|+\prod_{i=1}^{n}\left|x_{i}\right|$	30	[−10, 10]	0
$F_{3}(x)=\sum_{i=1}^{n}\left[x_{i}^{2}-10\cos(2\pi x_{i})+10\right]$	30	[−5.12, 5.12]	0
$F_{4}(x)=-20\exp\left(-0.2\sqrt{\frac{1}{n}\sum_{i=1}^{n}x_{i}^{2}}\right)-\exp\left(\frac{1}{n}\sum_{i=1}^{n}\cos2\pi x_{i}\right)+20+e$	30	[−32, 32]	0
$F_{5}(x)={\sum_{i=1}^{11}\left[a_{i}-\frac{x_{1}(b_{i}^{2}+b_{i}x_{2})}{b_{i}^{2}+b_{i}x_{3}+x_{4}}\right]}^{2}$	4	[−5, 5]	0.0003075
$F_{6}(x)=-{\sum_{i=1}^{5}\left[\left(x-a_{i}\right){\left(x-a_{i}\right)}^{T}+c_{i}\right]}^{-1}$	4	[0, 10]	−10

**Table 5:** The comparison results of each algorithm on the test functions.

Test Function	Statistical Value	PSO	GWO	WOA	IMWOA	TIWOA	IWOA
*F*_1_	Best	0.0613	1.02 × 10^−29^	8.27 × 10^−87^	1.86 × 10^−112^	1.78 × 10^−126^	**0**
	Std.	0.3739	1.23 × 10^−27^	8.84 × 10^−71^	2.21 × 10^−96^	3.28 × 10^−118^	**0**
	Avg.	0.3343	9.03 × 10^−28^	1.63 × 10^−71^	4.47 × 10^−97^	9.48 × 10^−119^	**0**
*F*_2_	Best	0.0223	1.71 × 10^−17^	1.86 × 10^−57^	4.85 × 10^−69^	2.68 × 10^−76^	**1.65 × 10^−282^**
	Std.	1.8291	6.69 × 10^−17^	1.79 × 10^−52^	1.05 × 10^−59^	1.16 × 10^−70^	**0**
	Avg.	0.4125	1.02 × 10^−16^	7.89 × 10^−53^	2.12 × 10^−62^	2.62 × 10^−71^	**2.56 × 10^−272^**
*F*_3_	Best	2.9887	5.68 × 10^−14^	0	0	0	**0**
	Std.	3.0255	4.13 × 10^−11^	2.08 × 10^−11^	8.82 × 10^−56^	2.14 × 10^−49^	**0**
	Avg.	3.3261	7.69 × 10^−11^	3.79 × 10^−12^	4.54 × 10^−55^	0.39 × 10^−50^	**0**
*F*_4_	Best	0.0889	5.73 × 10^−12^	2.89 × 10^−10^	3.96 × 10^−12^	6.47 × 10^−15^	**4.02 × 10^−16^**
	Std.	0.6303	2.02 × 10^−9^	2.38 × 10^−6^	2.48 × 10^−11^	1.60 × 10^−13^	**0**
	Avg.	0.707	1.05 × 10^−10^	3.88 × 10^−5^	8.40 × 10^−12^	3.76 × 10^−13^	**4.13 × 10^−16^**
*F*_5_	Best	3.24 × 10^−3^	3.10 × 10^−4^	3.23 × 10^−4^	3.41 × 10^−4^	4.81 × 10^−4^	**3.07 × 10^−4^**
	Std.	0.0115	4.63 × 10^−5^	1.00 × 10^−5^	0.0029	5.17 × 10^−4^	**5.38 × 10^−5^**
	Avg.	0.0071	3.64 × 10^−4^	4.23 × 10^−4^	0.002	5.27 × 10^−4^	**3.32 × 10^−4^**
*F*_6_	Best	−5.1532	−8.1529	−9.1516	−9.6113	−9.2465	**−9.8205**
	Std.	3.4034	1.2819	2.8456	1.94 × 10^−3^	0.1359	**7.23 × 10^−15^**
	Avg.	−3.1557	−9.2145	−7.7718	−9.6514	−8.9993	**−9.6723**

**Table 6:** Determination of the number of hidden layer nodes.

Number of Hidden Layer Nodes	RMSE	Number of Hidden Layer Nodes	RMSE
3	0.0953	8	0.0538
4	0.0756	9	0.0696
5	0.0885	10	0.0881
6	0.0342	11	0.1042
7	0.1483	12	0.1159

**Table 7:** Prediction results of *Ra* by different neural network models.

No.	True Values	BP Predicted Values	WOA-BP Predicted Values	IMWOA-BP Predicted Values	TIWOA-BP Predicted Values	IWOA-BP Predicted Values
1	2.407	2.672	2.620	2.328	2.505	2.388
2	1.445	1.760	1.186	1.507	1.367	1.471
3	1.136	0.852	1.001	1.259	1.199	1.124
4	2.036	2.168	2.133	1.935	1.933	2.027
5	1.240	0.873	1.546	1.312	1.299	1.245
6	2.354	2.065	2.162	2.269	2.246	2.298
7	2.120	2.327	2.207	2.209	2.056	2.157
8	1.802	1.386	2.025	1.725	1.880	1.851
9	1.568	1.731	1.314	1.485	1.506	1.510
10	2.236	2.490	2.405	2.369	2.328	2.249

**Table 8:** Prediction results of *Wa* by different neural network models.

No.	True Values	BP Predicted Values	WOA-BP Predicted Values	IMWOA-BP Predicted Values	TIWOA-BP Predicted Values	IWOA-BP Predicted Values
1	4.267	3.828	4.629	4.103	4.180	4.313
2	2.527	2.872	2.284	2.614	2.359	2.441
3	1.961	2.358	2.350	2.096	2.104	2.028
4	3.660	4.084	3.955	3.574	3.859	3.736
5	1.895	1.467	1.534	1.786	1.968	1.882
6	4.158	3.624	4.345	4.080	4.004	4.103
7	3.862	4.435	4.138	3.930	3.944	3.928
8	3.115	2.905	2.804	3.216	2.931	3.072
9	2.450	2.948	2.236	2.292	2.496	2.439
10	3.783	4.349	4.168	3.936	3.858	3.840

**Table 9:** Evaluation indicators of the prediction performance of different neural network models.

	Error type	BP	WOA-BP	IMWOA-BP	TIWOA-BP	IWOA-BP
Roughness *Ra*	RMSE	0.2816	0.2052	0.0929	0.0824	0.0342
	MAE	0.2692	0.1935	0.0904	0.0805	0.0284
	MAPE	16.0771%	11.6491%	5.2491%	4.4836%	1.5614%
Waviness *Wa*	RMSE	0.4537	0.3099	0.1188	0.1316	0.0570
	MAE	0.4414	0.3023	0.1139	0.1211	0.0520
	MAPE	14.8065%	10.5587%	3.9645%	4.0863%	1.7028%

## Data Availability

The original contributions presented in this study are included in the article. Further inquiries can be directed to the corresponding author.

## Abbreviations

The following abbreviations are used in this manuscript:

BP	Backpropagation
BBD	Box–Behnken design
WOA	Whale optimization algorithm
IWOA	Improved whale optimization algorithm
TIWOA	Threshold improved whale optimization algorithm
IMWOA	Improved multi-objective whale optimization algorithm
CIWOA	Chaotic improved whale optimization algorithm
WOA-BP	Whale optimization algorithm backpropagation
IWOA-BP	Improved whale optimization algorithm backpropagation
TIWOA-BP	Threshold improved whale optimization algorithm backpropagation
IMWOA-BP	Improved multi-objective whale optimization algorithm backpropagation
RMSE	Root mean square error
MSE	Mean square error
MAE	Mean absolute error
MAPE	Mean absolute percentage error
